# Synthesis and Purification of [Eu(BA)_4_(pip)] Rare-Earth Molecular Crystals

**DOI:** 10.3390/nano15171348

**Published:** 2025-09-02

**Authors:** Xiangtai Xi, Wenli Fan, Jun Huang, Haoyang Chen, Huan Chen, Zhengkun Fu, Zhenglong Zhang

**Affiliations:** School of Physics and Information Technology, Shaanxi Normal University, Xi’an 710062, China; xixiangtai@snnu.edu.cn (X.X.); wenli_fan@snnu.edu.cn (W.F.); jhuang@snnu.edu.cn (J.H.); hychen@snnu.edu.cn (H.C.); zlzhang@snnu.edu.cn (Z.Z.)

**Keywords:** single-crystal thin films, europium mononuclear complexes, organic molecular crystals, lattice structure, fluorescence emission spectra

## Abstract

Europium mononuclear complexes are able to form organic molecular crystals by aggregation of molecules through non-covalent bonding interactions. These crystals have many unique optical properties. However, this kind of crystal still faces some difficulties and challenges in the process of research and application, such as the high difficulty of synthesis and purification, and the difficulty of spectral property modulation. In this work, an europium-containing rare-earth molecular crystal material [Eu(BA)_4_(pip)], was prepared via a solvothermal method. It is characterized by low melting point, low polarity, stable structure, high luminescence intensity, and has the potential for the preparation of quantum optical devices. After that, optimized the structure of the molecular crystals by petroleum ether solvent. Through the recrystallization process, a uniform and continuous film was formed, which resulted with a more regular surface morphology, and the changes in the optimized crystal structure had an effect on the europium ion electron-leap energy level, the fluorescence emission spectra also showed higher fluorescence resolving ratio. This study particular emphasis on enhancing the quality of [Eu(BA)_4_(pip)] molecular crystals and investigating their impact on their spectral properties.

## 1. Introduction

Organic molecular crystals are solid-state materials formed by the orderly arrangement of small organic molecules in three-dimensional space through non-covalent interactions such as hydrogen bonding, van der Waals forces, and π–π stacking [1,2,3]. Compared with inorganic crystals, organic molecular crystals typically exhibit lower melting points, softer mechanical properties, and greater chemical tunability [4]. These features enable their widespread application in fields such as optoelectronic materials, pharmaceuticals, nonlinear optics, and organic semiconductors [5].

Rare-earth organic molecular crystals are generally formed through coordination interactions between rare-earth ions and organic ligands, with the coordination environment of the rare-earth ions playing a crucial role in determining the physical and chemical properties of the resulting crystals [6]. Common lattice structures of rare-earth organic molecular crystals include mononuclear complexes [7,8], polynuclear complexes [9,10,11], coordination polymers, and metal–organic frameworks (MOFs) [12], encompassing diverse architectures such as Claran-type structures, layered structures, and cage-like structures [13,14,15]. The structural diversity of these crystals imparts a wide range of physicochemical properties and functional potentials. By rationally designing and selecting appropriate ligands, the structure and properties of these crystals can be precisely tuned at the molecular level, offering significant opportunities for the development of novel functional materials.

The influence of lattice structure on doped rare-earth ion luminescent centers is a key topic in solid-state physics and materials science [16,17]. Critical factors affecting this influence include lattice matching, crystal field effects, the band structure of the host material, as well as defect states and impurities [18,19]. Investigating how the lattice structure impacts rare-earth ion luminescence centers not only deepens our understanding of the underlying emission mechanisms but also provides guidance for the design of novel luminescent materials with enhanced efficiency and stability [20,21,22].

The Xiamen Rare Earth Materials Research Center of the Fujian Institute of Physical Chemistry, Chinese Academy of Sciences, has reported a new design strategy for high-performance blue organic luminescent materials, and has developed materials with high luminous efficiency, nanosecond-level luminescence lifetime, and effective inhibition of concentration quenching [23]. Research by Zhai’s group at Huazhong University of Science and Technology has demonstrated that two-dimensional rare-earth compound Er_2_O_2_S exhibits intrinsic room-temperature magnetically induced circularly polarized luminescence [24]. This discovery is significant for advancing the application of 2D materials in spintronics. Research by Zou’s group dynamic chiral Eu (III) complexes exhibiting aggregation-induced emission and strong circularly polarized luminescence (CPL) were reported in 2024, underscoring how molecular packing can boost solid-state color purity [25]. Baitalik’s group reported the work on thermosensing/thermochromism illustrates the translational potential of Eu (III) β-diketonate platforms, complementing the application outlook [26]. Due to their unique optical, magnetic, and electronic properties, two-dimensional rare-earth materials hold promising potential for next-generation lighting technologies, magnetic devices, and optoelectronic transistors [27,28,29].

In this study, we synthesized a novel rare-earth molecular crystal, [Eu(BA)_4_(pip)], by employing a solvothermal method that combines the advantages of rare-earth elements and organic molecular crystals. The luminescent properties of the material were thoroughly investigated. Additionally, molecular crystal thin films were fabricated, and the influence of lattice structural variations on their spectral characteristics was explored. The results reveal that the thin film exhibits a more ordered molecular arrangement compared to the bulk crystal, along with a greater number of distinct fluorescence emission peaks. This represents a new class of rare-earth molecular crystal thin films.

## 2. Materials and Methods

### 2.1. Experimental Instruments and Reagent

The main instruments used in this study include a magnetic stirrer (MR Hei-Tec, Hechen Technology Co., Ltd., Shanghai, China), an electronic balance (SQP, Sartorius, Niedersachsen, Gottingen, Germany), an ultrasonic cleaner (DR-MS30, Derui Precision Co., Ltd., Shenzhen, China), a circulating water vacuum pump (SHB-III, Zhengzhou Greatwall Scientific Industrial and Trade Co., Ltd., Zhengzhou, China), an electric blast drying oven (Model 101, Beijing Kewei Yongxing Instrument Co., Ltd., Beijing, China), and a multi-head magnetic heating stirrer (HJ-4A, Jiangsu Jinyi Instrument Technology Co., Ltd., Changzhou, China). Surface morphology of the samples was characterized using a field emission scanning electron microscope (FESEM) from Nava NanoSEM 450 (Thermo Fisher Scientific Inc., Waltham, MA, USA) and a field emission transmission electron microscope (FETEM) from Talos F200i (Thermo Fisher Scientific Inc., Waltham, MA, USA). X-ray diffraction (XRD) measurements were carried out using an instrument manufactured by D8 DISCOVER A25 (Bruker, Walzbachtal, Berlin, Germany). Fourier transform infrared (FTIR) spectra were obtained using a spectrometer from Vertex70 (Bruker, Walzbachtal, Berlin, Germany). The spectrum were carried out using a high resolution microscopic confocal Raman spectrometer (LabRAM HR Evolution, HORIBA JOBIN YVON S.A.S., Paris, France).

Experimental reagents required for the preparation of [Eu(BA)_4_(pip)] molecular crystals and their thin films: Benzoylacetone (C_10_H_10_O_2_, 98+%, Alfa Aesar, Shanghai, China); Europium (III) Chloride Hexahydrate (EuCl_3_·6H_2_O, 99.9%, Sigma-Aldrich, Shanghai, China); Piperidine (C_5_H_11_N, 99.5%, Sinopharm Chemical Reagent Co., Ltd., Shanghai, China); Ethanol absolute (C_2_H_6_O, 99.5%, Sinopharm Chemical Reagent Co., Ltd., Shanghai, China); Petroleum ether (C_6_H_6_, 99.5%, Sinopharm Chemical Reagent Co., Ltd., Shanghai, China); Conductive glue (AGG3939, Agar Scientific, Oxford Instruments, Shanghai, China).

### 2.2. Preparation of Rare-Earth Molecular Crystals

The [Eu(BA)_4_(pip)] molecular crystal was synthesized via a solvothermal method [30]. Specifically, 1.3 g (8 mmol) of benzoylacetone (BA) was dissolved in 20 mL of ethanol and stirred on a magnetic stirrer at 75 °C until fully dissolved, forming the ligand solution. Subsequently, 0.8 mL (8 mmol) of piperidine (pip) was added and stirred for an additional 15 min. Then, 732.64 mg (2 mmol) of europium(III) chloride hexahydrate (EuCl_3_·6H_2_O), dissolved in 10 mL of deionized water, was added to the reaction mixture. After turning off the heat, the solution was allowed to cool naturally to room temperature and stirred continuously for 24 h, yielding a light pink microcrystalline precipitate. The resulting precipitate was separated from excess organic solvents by filtration. The precipitate was washed several times with 50 mL of ethanol, and the filtered solid was dried overnight at 60 °C in a drying oven to obtain the final product.

### 2.3. Preparation of Molecular Crystal Thin Films

**Figure 1:**
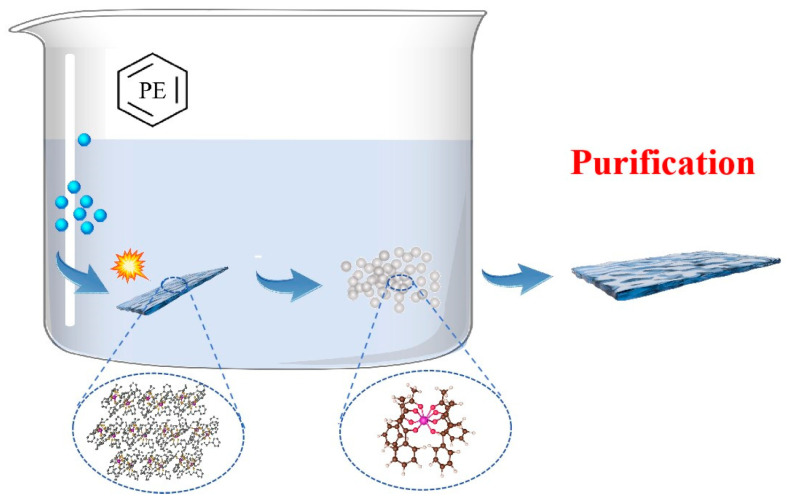
Synthesis and Purification of [Eu(BA)_4_(pip)].

First, an accurate amount of 0.01 g of [Eu(BA)_4_(pip)] molecular crystals was weighed and dissolved in 20 mL of petroleum ether. Petroleum ether was selected as the solvent due to its effectiveness in dissolving rare-earth organic molecular crystals and its high volatility, which facilitates the subsequent recrystallization process. To ensure thorough dissolution, the solution was stirred carefully using a magnetic stirrer. To further improve dissolution efficiency and ensure homogeneity, the solution was treated with an ultrasonic cleaner. Once fully dissolved, the solution was drop-cast onto a thoroughly cleaned and pretreated silicon wafer using a rubber-tipped dropper. As the solvent gradually evaporated, [Eu(BA)_4_(pip)] molecular crystals began to precipitate on the wafer surface, forming a uniform and continuous thin film. Finally, the solvent was completely removed either through natural evaporation or by applying mild heating at 50 °C for 2 h, resulting in the formation of a [Eu(BA)_4_(pip)] molecular crystal thin film. As shown in Figure 1.

## 3. Results and Discussion

**Figure 2:**
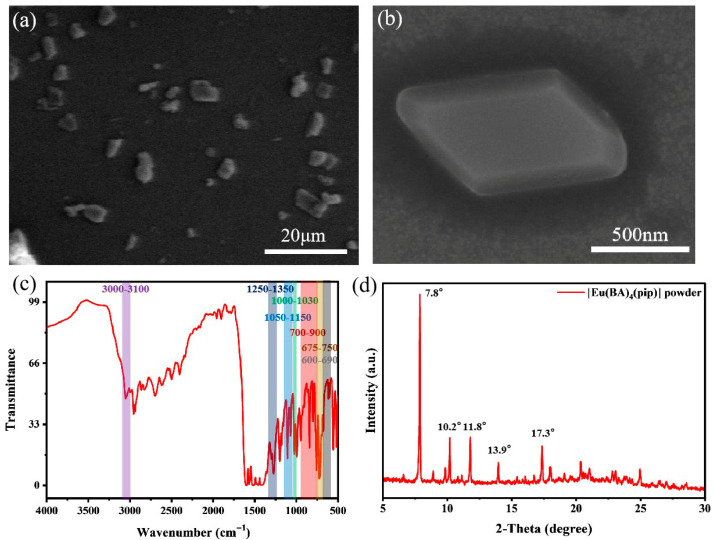
The Characterization of [Eu(BA)_4_(pip)] molecular crystal. (**a**) The FESEM image of [Eu(BA)_4_(pip)] molecular crystal. (**b**) The enlarged view of a single block-shaped crystal in (**a**). (**c**) FTIR spectroscopy of [Eu(BA)_4_(pip)] molecular crystal. (**d**) XRD pattern of [Eu(BA)_4_(pip)] molecular crystal.

In this work, the solvothermal synthesis method was used to prepare the coordination compound [Eu(BA)_4_(pip)] molecular crystals containing europium ions. A small amount of powder was picked up with a wooden stick and evenly spread on the conductive glue, and then the conductive glue was adhered to the sample platform. The prepared samples were characterized using FESEM, and the results are shown in Figure 2a,b. From the figures, it can be seen that each [Eu(BA)_4_(pip)] molecular crystal presents a relatively regular polyhedral shape similar to the orthorhombic crystal system, with a smooth surface and no obvious cracks, and the long diagonal is approximately 1.5 μm. This is in good agreement with previous reports, where the same material was synthesized with similar polyhedral morphology [31]. The differences in size may be attributed to variations in select samples.

FTIR spectroscopy provides insight into molecular vibrational modes, enabling the identification of chemical bonds and functional groups within a material. The results are shown in Figure 2c. The [Eu(BA)_4_(pip)] complex was synthesized using piperidine and benzoylacetone as ligands, and the observed infrared absorption bands are directly correlated with functional groups present in these ligands. Absorption peaks in the 3100–3000 cm^−1^ region are typically attributed to the C–H stretching vibrations of aromatic and cyclic structures, which in this case correspond to aromatic C–H bonds in both the piperidine ring and the benzoylacetone moiety [32,33]. The absorption bands in the range of 1250–1350 cm^−1^ arise from in-plane bending vibrations of aromatic C–H bonds and C–N stretching vibrations within the piperidine ring [32,34]. Additional characteristic peaks around 1000–1030 cm^−1^ are assigned to the ring-breathing modes of piperidine, while the broader region from 1000 to 1300 cm^−1^ also includes contributions from C–H in-plane bending in the aromatic rings and possible weak absorptions from C–O single bond stretching (1050–1150 cm^−1^) [32,35]. In the 600–900 cm^−1^ region, absorptions are attributed to the out-of-plane C–H bending of the benzoylacetone aromatic ring (700–900 cm^−1^), in-plane C–H bending of piperidine (675–750 cm^−1^), and out-of-plane bending modes of the piperidine C–H bonds (600–690 cm^−1^) [31,32]. These observations confirm that the functional groups present in the synthesized [Eu(BA)_4_(pip)] molecular crystal correspond well with those of the benzoylacetone and piperidine ligands.

The synthesized [Eu(BA)_4_(pip)] microcrystalline powder was characterized by XRD, and the results are shown in Figure 2d. The XRD pattern exhibits distinct diffraction peaks at 2θ values of 7.8°, 10.2°, 11.8°, 13.9°, and 17.3°. After comparing with the data reported by Diana Serrano et al., we reached the conclusion that the peak positions are basically the same [31]. The relative intensities of the diffraction peaks are also comparable, indicating structural similarity. The sharp and narrow full width at half maximum (FWHM) of the characteristic peaks suggests that the synthesized [Eu(BA)_4_(pip)] crystals possess high crystallinity. The crystal structure was further analyzed and identified as belonging to the monoclinic system with a space group of P2_1_/n. Together with the XRD data, the FTIR results validate the successful synthesis of the target complex.

**Figure 3:**
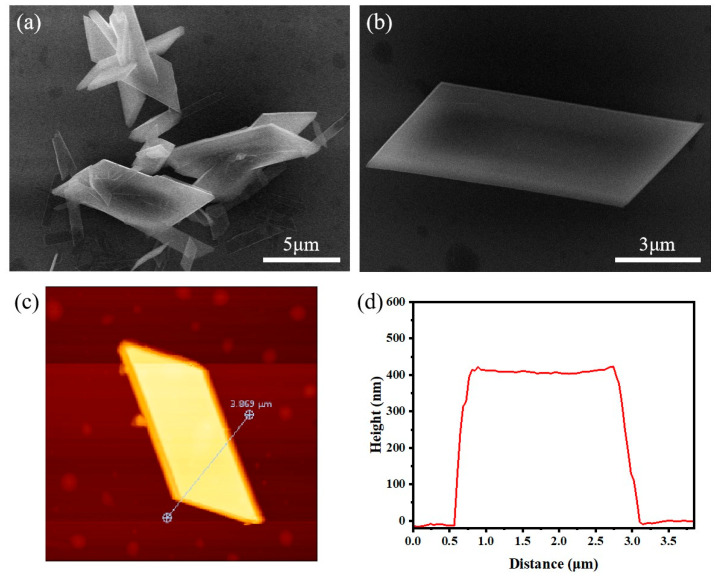
The Characterization of [Eu(BA)_4_(pip)] molecular crystal thin films after purification. (**a**) The FESEM image of [Eu(BA)_4_(pip)] molecular crystal thin films. (**b**) The enlarged view of a single crystal in (**a**). (**c**) The AFM images of the [Eu(BA)_4_(pip)] molecular crystal thin films. (**d**) The surface height map of (**c**).

Precise design of the lattice structure enables optimization of the luminescent properties of rare-earth doped materials, influencing factors such as site symmetry and energy transfer efficiency, thereby providing a crucial materials foundation for the development of efficient phosphors, display technologies, and lasers [36,37]. The SEM images of the [Eu(BA)_4_(pip)] molecular crystal thin films are shown in Figure 3a,b. Compared to the original crystals in Figure 2a,b, the recrystallized thin films obtained after petroleum ether treatment exhibit significant morphological improvements, with crystal sizes ranging from 10 to 30 μm. The surface of the recrystallized films is smoother, and the crystal edges are more well-defined, indicating that the recrystallization process effectively reduces surface defects and irregularities. Notably, these crystals display highly regular parallelogram shapes with well-defined edges and uniform dimensions, indicating a high degree of geometric symmetry. Atomic force microscope (AFM) images of the [Eu(BA)_4_(pip)] molecular crystal thin films are presented in Figure 3c, with the surface height map shown in Figure 3d. The film thickness is approximately 400 nm, with lateral dimensions of about 5 μm by 3 μm. The smooth surface and absence of obvious cracks indicate superior structural stability and uniformity. The clear edges observed in the AFM images further emphasize the highly ordered structure formed during crystal growth. Collectively, these features highlight the excellent surface quality and structural integrity of the [Eu(BA)_4_(pip)] molecular crystal thin films, suggesting their promising potential for optoelectronic applications.

**Figure 4:**
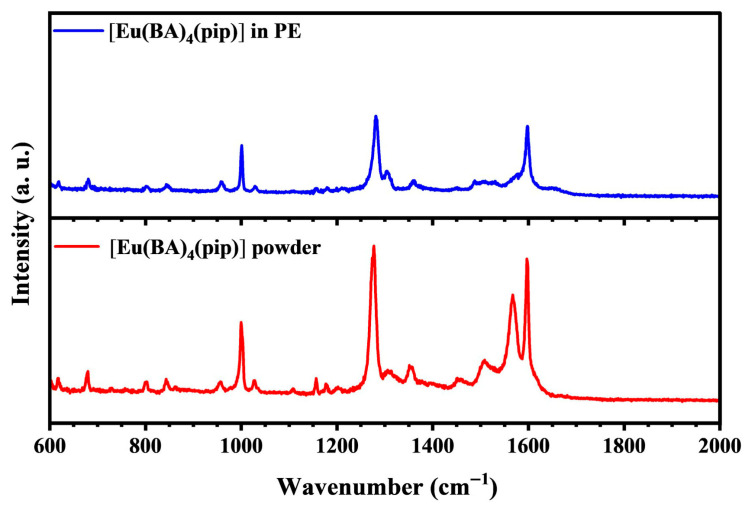
The Raman spectrum of the [Eu(BA)_4_(pip)] molecular crystal, excited with a 633 nm laser. The red curve corresponds to the experimentally measured Raman spectrum of the original molecular crystals, and the blue curve shows the Raman spectrum of the recrystallized thin films obtained after petroleum ether treatment.

The Raman spectrum of the [Eu(BA)_4_(pip)] molecular crystal, excited with a 633 nm laser, is presented in Figure 4. The red curve corresponds to the experimentally measured Raman spectrum of the original molecular crystals, and the blue curve shows the Raman spectrum of the recrystallized thin films obtained after petroleum ether treatment. The absorption band at 1000 cm^−1^ corresponds to the stretching vibration between the benzene ring and carbon atoms, the band at 1300 cm^−1^ corresponds to the stretching vibration between methyl and carbon atoms, and the band at 1600 cm^−1^ corresponds to the stretching vibration between oxygen and carbon atoms. During the recrystallization process, petroleum ether primarily functions to reduce impurities. It not only dissolves the target compound but also selectively removes impurities by differential solubility. Impurities insoluble in petroleum ether are effectively separated, thereby enhancing the purity of the final product. Additionally, the use of petroleum ether influences crystal morphology and growth habits. In certain cases, the addition of petroleum ether promotes the formation of crystals with specific facets, which is particularly important for applications requiring well-defined crystal morphologies, such as in optical and electronic devices. A careful comparison of the Raman spectra before and after recrystallization reveals several notable changes, although the peak positions remain largely unchanged. Specifically, some Raman peaks in the recrystallized samples become weaker and the overall spectral profile appears smoother. This phenomenon is likely attributed to an increase in the structural order of the crystal induced by the recrystallization process, which effectively reduces defects both on the crystal surface and within the bulk. During recrystallization, petroleum ether acts as a mild and controllable solvent environment that facilitates more ordered molecular packing and defect healing during crystal growth, resulting in significantly improved crystal quality. This enhanced structural order not only alleviates microscopic stresses inside the crystals but may also influence their optical properties, for instance by reducing non-radiative recombination losses and thus improving luminescence efficiency. Therefore, the changes observed in the Raman spectra of the [Eu(BA)_4_(pip)] molecular crystals after petroleum ether recrystallization directly reflect the enhancement of crystal quality and optimization of crystal structure, which are of critical importance for understanding the material’s physical properties and potential applications.

The crystal field effect refers to the influence of the electric field generated by interactions among ions, atoms, or molecules within a crystal on the electronic energy levels of ions. In solid-state physics and chemistry, this effect primarily concerns transition metal and rare-earth ions, whose partially filled d or f orbitals experience splitting of their energy levels under the electric field created by surrounding ions or molecules.

**Figure 5:**
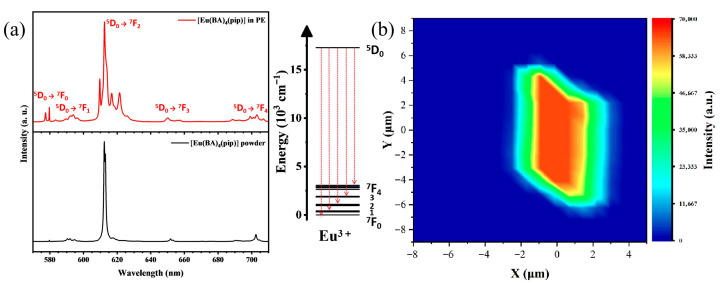
The optoelectronic devices of [Eu(BA)_4_(pip)] molecular crystal and thin films. (**a**) The comparative emission spectra. The attached figure is the energy level diagram of Eu^3+^. (**b**) The fluorescence mapping image of the [Eu(BA)_4_(pip)] molecular crystal thin films.

The photoluminescence emission spectrum of [Eu(BA)_4_(pip)] was also analyzed, as shown in Figure 5a. Distinct emission peaks corresponding to the electronic transitions of Eu^3+^ from the excited state ^5^D_0_ to the ^7^F_J_ levels (J = 0, 1, 2, 3, 4) were observed. Among these, the sharp peak near 612 nm is the most intense, corresponding to the ^5^D_0_ → ^7^F_2_ transition, which is characteristic of a strong Stokes shift and an electric-dipole-allowed transition. The intensity and sharpness of the emission peaks provide insight into the quantum efficiency and non-radiative relaxation processes of the complex. The high emission intensity and narrow peak widths indicate low non-radiative relaxation rates and high quantum efficiency, which are attributed to efficient energy transfer from the ligands to Eu^3+^ and effective shielding of the luminescent center. The choice of ligand and coordination environment plays a critical role in modulating the emission behavior. Specific ligands not only enhance Eu^3+^ luminescence via ligand-to-metal energy transfer mechanisms but also influence the peak positions and spectral profiles through their molecular structures and electronic properties. The emission spectral analysis of [Eu(BA)_4_(pip)] reveals its excellent optoelectronic performance and the presence of a complex energy transfer mechanism.

Fluorescence emission spectra of [Eu(BA)_4_(pip)] molecular crystal thin films were measured using a microscopic confocal Raman spectrometer under 532 nm excitation. The comparative emission spectra are shown in Figure 5a, where the black curve represents the fluorescence spectrum of the original molecular crystals and the red curve corresponds to that of the recrystallized thin films. The comparison reveals notable spectral changes after petroleum ether recrystallization, including peak splitting, variations in peak widths, and relative intensity changes, reflecting the impact of altered crystal environments on the luminescent properties of the Eu^3+^ ions. During recrystallization, subtle modifications in the internal crystal structure likely alter the coordination environment surrounding the europium ions, leading to splitting or shifts in specific energy levels. For example, the originally observed strong, split peak near 612 nm evolves into four distinct split peaks with unchanged overall intensity. This indicates an enhanced crystal field effect on the Eu^3+^ ions, likely caused by changes in crystal symmetry induced by recrystallization, which modifies the local crystal field environment of the ions. Such modifications promote more detailed splitting of energy levels, demonstrating that the europium ions experience varying degrees of crystal field interaction. Through recrystallization of the europium-based rare-earth molecular mononuclear complex in petroleum ether, subtle alterations in both the crystal lattice and coordination environment occur. These are manifested in the fluorescence spectra as energy level splitting, enhanced relative intensities, peak broadening, and fine structure splitting, all indicative of changes in the crystal field effect and its influence on the Eu^3+^ electronic energy levels and transition probabilities. These spectral changes not only provide critical insights into the internal structural modifications of the crystal but also bear significant implications for the material’s optical properties and potential applications. These spectral changes can have significant consequences for optoelectronic device applications. For example, peak shifts and intensity enhancement can improve the color purity and external quantum efficiency of light-emitting diodes (LEDs). Similarly, controlled emission wavelength and improved luminescence intensity are advantageous for optical amplifiers and laser devices, as they enable better gain spectral matching and lower lasing thresholds. Moreover, for applications in upconversion devices or single-photon sources, the structural tuning may enhance energy transfer efficiency and emission stability.

The fluorescence mapping image of the [Eu(BA)_4_(pip)] molecular crystal thin films under 532 nm excitation is shown in Figure 5b. The fluorescence mapping was performed over a single crystal area of 20 × 15 μm^2^, focusing on the spatial distribution of fluorescence characteristics within the crystal, including variations in intensity and color, to reveal the homogeneity of fluorescence, presence of defect regions, and their spatial correlations. By precisely controlling the mapping area and utilizing high-resolution observation, localized variations in fluorescence intensity as well as subtle differences in peak position and shape across different regions were identified. These observations provide critical insights into the heterogeneity of the electronic structure and energy level distribution within the single crystal. Furthermore, they may indicate how physical properties such as crystal orientation, defect types, and doping levels influence the fluorescence behavior. Under 532 nm excitation, the emission intensity at 612 nm was found to be uniformly distributed in the central region of the single crystal, suggesting a defect-free surface and homogeneous fluorescence distribution. This uniformity supports in-depth investigations of the material’s optoelectronic properties and provides a solid basis for optimizing its applications in optoelectronic devices, bioimaging, and related fields.

The structural transformation of [Eu(BA)_4_(pip)] organic molecular crystals in petroleum ether can be explained by several possible mechanisms: 1. Solvent Effects: Polarity and Solubility. Petroleum ether is a low-polarity solvent, and its solubility behavior toward organic molecular crystals depends on the interplay between solvent polarity and solute polarity. Changes in the solubility of the europium complex in petroleum ether may alter intermolecular interactions among solute molecules, leading to molecular rearrangements or structural transformations. Additionally, a solvent cage effect may occur, where solvent molecules surround solute species and influence their behavior. In petroleum ether, such effects could induce adjustments in the mononuclear structure of the europium complex during dissolution; 2. Changes in Coordination Environment. Many europium complexes exhibit coordination dynamics in solution, where ligands can exchange or rearrange at coordination sites. During dissolution, petroleum ether may modify the local microenvironment around the complex, promoting ligand re-coordination and thereby causing structural changes. Moreover, solvent molecules in petroleum ether might participate in the coordination sphere, forming new bonds with Eu^3+^ ions and leading to transformations from the original mononuclear complex structure; 3. Crystal–Solution Interactions. The europium complex crystals may partially or fully dissociate during dissolution, which can disrupt the original crystal structure and facilitate the formation of new phases. Recrystallization can also occur, especially when the solution approaches saturation, resulting in the formation of crystals with different morphologies or structures; 4. Influence of Temperature and Pressure. Variations in temperature and pressure during the dissolution process can further impact structural transformations. Changes in temperature can affect solubility, coordination environment, and crystal growth kinetics, all of which may contribute to lattice rearrangement or phase transitions [38,39].

In summary, petroleum ether plays an active role in the purification of [Eu(BA)_4_(pip)] organic molecular crystals by influencing their solubility, coordination dynamics, and recrystallization behavior, thereby facilitating structural transformations beneficial for achieving high-quality crystals.

## 4. Conclusions

This study primarily focuses on the development and optimization of rare-earth organic molecular crystals for high-performance optoelectronic applications, with particular emphasis on enhancing the quality of [Eu(BA)_4_(pip)] molecular crystals through an improved recrystallization method and investigating its impact on their spectral properties. Through meticulous experimental design, we not only elucidated the critical role of petroleum ether as a solvent in the crystal growth process but also revealed the profound influence of crystal structure on the optical spectra. These findings provide important scientific insights and technical approaches for the development of novel high-performance rare-earth-based optoelectronic materials, laying a solid foundation for their future applications in photodetection, information storage, and bioimaging fields. Future research on rare-earth organic molecular crystals may focus on material design through the combination of diverse rare-earth ions and ligands, optimization of crystal growth conditions to improve crystallinity and reduce defects, and systematic studies of their optoelectronic properties using advanced characterization techniques. Application development in luminescent devices, optoelectronic sensors, and bioimaging is particularly promising.
